# Barriers and facilitators to accessing healthcare for early diagnosis of prostate cancer for black men—a qualitative exploration in North-East England and Scotland

**DOI:** 10.1186/s12889-025-23650-y

**Published:** 2025-07-14

**Authors:** Floor Christie-de Jong, Olugbenga Samuel Oyeniyi, Lawrence Achilles Nnyanzi, Jonathan Ling, Marie K. Murphy, Judith Eberhardt, Rawand Jarrar, John Kabuye, Martin Kalemba, Kathryn A. Robb

**Affiliations:** 1 School of Medicine, Faculty of Health Sciences and Wellbeing, University of Sunderland, Sunderland, UK; 2 School of Health & Life Sciences, Teesside University, Middlesbrough, UK; 3 Darlington, UK; 4 School of Medicine, Dentistry, and Nursing, Dental School, University of Glasgow, Glasgow, UK; 5 School of Social Sciences, Humanities and Law, Department of Psychology, Teesside University, Middlesbrough, UK; 6 School of Health & Wellbeing, University of Glasgow, Glasgow, UK

**Keywords:** Black men, Prostate cancer, Health inequalities, Barriers, Qualitative, Racism

## Abstract

**Introduction:**

Prostate cancer is the most commonly diagnosed cancer in men in the United Kingdom. There are substantial inequalities in prostate cancer, with Black African and Caribbean men at least twice as likely as White men to develop prostate cancer, and twice as likely to die from it. Black men need to be aware of their elevated risk, which can encourage help-seeking behaviour leading to early diagnosis. This study aimed to investigate barriers and facilitators to accessing healthcare for early diagnosis of prostate cancer for Black men.

**Methods:**

Barriers and facilitators were explored through online focus groups with Black men (*n* = 13) from Scotland and North-East England, who formed the Public Involvement and Community Engagement group for a larger study. Purposive and snowball sampling was used. Focus groups were audio-recorded and transcribed verbatim. Data analysis was iterative. Framework analysis was used and data were mapped onto the Integrated Screening Action Model (I-SAM).

**Results:**

Participants believed Black men lack prostate cancer knowledge. Additionally, prostate cancer communication needs to use language that Black men could identify with. Participants shared a lack of trust in healthcare providers and perspectives emerged resulting from negative healthcare experiences, including experiences of racism, as barriers to accessing healthcare for early prostate cancer diagnosis. Difficulties with accessing care, including navigating the healthcare system and making appointments, as well as cultural, social and religious factors, were reported as barriers to prostate cancer health checks. Discussing intimate and sensitive issues such as prostate cancer was perceived as difficult for Black men. The involvement of community and religious leaders, along with the collectivist characteristic of the community and the belief in staying healthy for the benefit of the family, were perceived as facilitators.

**Conclusion:**

Barriers to accessing healthcare for early prostate cancer diagnosis are complex and multifaceted, requiring complex solutions. Asset-based, participatory, and culturally tailored interventions have the potential to be effective in addressing barriers, and thus ultimately reduce morbidity and mortality through earlier diagnosis of prostate cancer in Black communities.

**Supplementary Information:**

The online version contains supplementary material available at 10.1186/s12889-025-23650-y.

## Introduction

Prostate cancer is the most common cancer among men in Europe and the United Kingdom (UK), accounting for 27% of all cancers among men in the UK with approximately 52,000 new prostate cancer cases every year [1, 2]. Prostate cancer is also the third most common cause of cancer death in the UK and Europe [1], accounting for 14% of all deaths from prostate cancer and 22% of all deaths among Black men [3]. Since the early 1990 s, incidence rates have almost doubled for all men, possibly due to increased access to prostate-specific antigen (PSA) testing, as well as a reflection of a population that lives longer but is ageing. It is estimated there could be around 85,000 new cases every year by 2038–2040 [3]. Inequalities exist in the prevalence, stage at diagnosis, and mortality of prostate cancer, with Black African and Caribbean men being at least twice as likely as White men to develop the disease and die from it [3–6]. Geographic variation is also evident, with significantly higher proportions of men being diagnosed with metastatic prostate cancer in Scotland and North -East England compared to Southern regions of the UK. This pattern reflects socioeconomic inequalities, with men in more deprived areas more likely to receive a late-stage diagnosis [7, 8]. These inequalities in prostate cancer outcomes for Black men are under-researched and unjust. We use the term ‘Black’ for simplicity, although we appreciate that this is a diverse group. African-Caribbean refers to people of African ancestry with origins from the Caribbean, who may identify as being of mixed heritage, Black British, Black American, or similar.

Early diagnosis of prostate cancer can lead to better treatment outcomes [9, 10]. Enhancing awareness of risk factors and signs and symptoms of prostate cancer could promote timely help-seeking behaviours, such as going to the doctor, discussing the risk of prostate cancer and/or asking for a prostate cancer health check, which can lead to earlier detection [11]. However, research suggests that only approximately one quarter of Black men are aware of their heightened risk [12]. Black men must be informed about the disease and their increased susceptibility to prostate cancer incidence and mortality. The EU and UK National Screening Committee do not recommend prostate screening although the EU has recently recommended further research to evaluate the feasibility and effectiveness of organised prostate cancer screening [13]. In the UK, all men over the age of 50 are entitled to ask for a PSA test, and Black men aged 45 and over are encouraged by the National Health Service (NHS) to talk about their risk with their doctor [14].

The literature shows barriers to early diagnosis of prostate cancer among Black men are intricate and multifaceted. They encompass various social, emotional, cultural, and structural barriers, and also insufficient knowledge of prostate cancer [6, 15–19]. For example, barriers such as communication and trust issues with healthcare providers, embarrassment, fear of the procedure and the outcome, or of being emasculated, have been described in the literature [15, 16]. Trying to overcome such barriers to accessing healthcare for early diagnosis could encourage help-seeking behaviours in this population. Most of the literature regarding barriers and facilitators to early diagnosis of prostate cancer originates from the United States. There is limited research in the UK on understanding barriers to early diagnosis of prostate cancer for Black men. A qualitative study with Black men (*n* = 18) to understand help-seeking and symptom appraisal following the onset of symptoms revealed a lack of awareness of prostate cancer and cultural stigma [20]. Understanding barriers and facilitators to early diagnosis of prostate cancer for UK-based Black men before the onset of symptoms is essential for addressing prostate cancer inequalities and may allow for improved outcomes in this group.

The present study is set in two socioeconomically deprived areas with poor cancer outcomes: the North-East of England and Scotland, which lack inclusion in UK research. Shaw et al.’s (2023) study was conducted in London, an ethnically diverse setting. Including Black men from less ethnically diverse settings is important to ensure their voices are heard too as these men may have different experiences accessing healthcare. This research therefore aimed to explore barriers and facilitators to early diagnosis of prostate cancer for Black men in the North-East of England and Scotland.

## Methods

### Study design

This study is reported using the Standards for Reporting Qualitative Research (SRQR) guidelines [21]. A phenomenological qualitative study using focus groups, nested within a larger mixed-method study to co-design an intervention, was conducted January-June 2023 to explore barriers and facilitators to early diagnosis of prostate cancer in Black men. This paper reports findings from this initial qualitative phase.

### Theoretical framework

This study draws on principles of the Integrated Screening Action Model (I-SAM) [20], which presents a comprehensive approach to understanding cancer screening behaviour and identifying areas for intervention. The I-SAM integrates three key components: (1) the stages of behaviour change from the Precaution Adoption Process Model [22]), (2) targets for behaviour change from the COM-B model [23], and (3) the interrelationships between individual, social and environmental factors of the socio-ecological model [24]. Although there is no prostate cancer screening programme in the UK yet, health behaviour related to early prostate cancer diagnosis resembles that of existing UK cancer screening programmes (e.g. breast, cervical or colorectal screening). Therefore, we believe the I-SAM enhances our understanding of health behaviours related to early diagnosis of prostate cancer. It provides a foundation for intervention development, and helps anticipate barriers and facilitators that may influence Black men’s engagement with a potential future prostate cancer screening programme.

### Sampling and recruitment

Two Recruitment Leads were appointed. They are members of the community and connected to community organisations. In true participatory style, they were employed as part of the research team, one in the North-East (JK) and one in Scotland (MKa). As a first step, and to inform the entire study, the Recruitment Leads established a Public Involvement and Community Engagement (PICE) group. Inclusion criteria focused on Black men aged 45 years or above and living in the target regions. As the study focused on healthy men engaging with prostate cancer health checks, no history of prostate cancer was required. While men with a personal history of prostate cancer were not excluded, none took part in the study. Purposive sampling was used to ensure multiple African ethnicities were included. However, snowball sampling was also applied. A recruitment advertisement, in the form of a flyer, was shared with community networks in both settings and recruitment was accomplished through word of mouth and social media (WhatsApp). The PICE group consisted of 13 Black men living in the North-East and Scotland combined; demographic characteristics are presented in Table 1.

**Table 1:** Sociodemographic characteristics of PICE group participants (*n* = 13)

		*N*
Location	North-East England	8
	Scotland	5
Age (years)	45–49	7
	50–54	3
	55–59	3
Religion	Christian	13
Marital Status	Married/domestic partnership	13
Highest education	GCSE or O levels	2
	Bachelor degree	4
	Master degree	7
Employment	Full time	7
	Part-time	1
	Self-employed	3
	Unemployed-looking for work	1
	Unemployed not looking for work	1
Disability	No	13
Main language at home	English	7
	Yoruba	2
	Bantu	1
	Nilotic	1
	Swahili	1
	French	1
Living arrangements	Own my home outright	2
	Own with a mortgage	5
	Rent from local authority	3
	Rent privately	3
Ethnicity	Black African	11
	Black Caribbean	2
Country of birth	United Kingdom	2
	Nigeria	4
	Uganda	2
	Cameroon	1
	Zambia	1
	Zimbabwe	1
	Sudan	1
	Congo	1
If not born in the UK, how long lived in the UK (years)	1–5	2
	6–10	0
	10+	9
Personal history of prostate cancer	No	13
Family history of prostate cancer	Yes	1
	No	12

### Data collection

Data were collected from a series of three focus groups with the PICE group members (*n* = 13). Attendance varied: eight men attended the first meeting, eleven the second, and seven the third. All 13 participants attended at least one meeting, and nine attended two or more. Although attendance varied slightly across sessions, the group dynamic remained consistent throughout, with a sustained sense of openness, trust, and active engagement. Initially, we had planned to conduct two focus group sessions only. However, as it was unclear whether data saturation had been fully achieved, a third session was conducted, which produced no new themes. Rather, the themes identified earlier were repeated and confirmed. Transcripts were not returned to participants; however, the final focus group was used as a member-checking exercise, where qualitative findings regarding barriers and facilitators were reported back to participants as a form of inside-outside legitimisation to enhance credibility and trustworthiness of the data [25]. This exercise confirmed that participants agreed with the analysis and additionally confirmed that data saturation had been reached and that no new codes or themes emerged. These focus groups marked the first time the PICE group members met. Time was needed for participants to become familiar with each other, establish rapport with the researchers, gradually open up and feel comfortable with discussing a sensitive and intimate topic such as prostate cancer checks. Repeating focus groups with the same sample is relatively uncommon but can be a valuable strategy to build trust with participants and explore issues in greater depth [25].

Each focus group lasted between 1.5 and 2 h. Focus groups were run approximately once a month, January-March 2023. JL led the first two focus groups and LN led the remaining focus group, with co-facilitation by OO and JK in all three sessions. JL and LN are both experienced researchers and had no prior engagement with participants. All facilitators were male and, apart from JL, all were Black. Focus groups were conducted online via Zoom, to accommodate participants from both regions. The online focus groups used a combination of large and smaller group discussions in break-out rooms. Smaller group discussions offered an opportunity for all members to contribute to discussions. All focus groups were audio-recorded and transcribed verbatim.

Semi-structured topic guides were created for each session. The initial topic guide was created based on barriers and facilitators found in the literature and carefully reviewed by the entire research team. The subsequent topic guides were adapted to explore in more depth issues raised in previous sessions or to explore areas not yet discussed (see supplementary files).

### Data analysis

Data analysis was iterative and conducted after each focus group, teasing out issues that required further exploration. Data were analysed using thematic analysis [26] by two female (FC & RJ), White, non-British researchers who are experienced in qualitative research. Researchers conducted coding independently using qualitative research software NVivo V12 [27], after which inductive coding was compared and discussed. Data were subsequently mapped onto the individual (capability, motivation) and environmental (opportunity) components of the Integrated Screening Action Model (I-SAM), which draws on the COM-B model, to inform the project’s next phase -intervention development. The framework of themes, subthemes and codes was discussed and checked with the focus groups’ facilitators and the larger research team on multiple occasions.

### Ethical considerations

Ethical approval was obtained from the University of Sunderland Research Ethics Committee (#015660) in December 2022. Participants were provided with a Participant Information Sheet, as well as an accessible study explanation offered in a short video, recorded by FC and OO. Written online consent was collected using Qualtrics. Each participant received a gift voucher worth £35 per focus group.

## Results

Results are discussed using the I-SAM model’s overarching structure: Individual Influences (capability and motivation) and Environmental Influences (opportunity).

### Individual influences

Individual-level influences encompassed ‘capability’ and ‘motivation’. Capability includes physical and psychological skills to engage in early diagnosis of prostate cancer, such as possessing prostate cancer knowledge, communication and language barriers, and lack of time. Motivation refers to prostate risk perception, emotions, like fear, experiences of racism and lack of trust in the healthcare system and healthcare providers.

### Capability

#### Knowledge and awareness

Knowledge and awareness of prostate cancer risk were discussed by all participants and perceived as a prerequisite for engaging in early diagnosis of prostate cancer. Not being aware of or having a lack of knowledge was perceived as an important barrier. Lack of knowledge was broken down into: (1) knowledge of health behaviours that were seen as conducive to health, such as acting on early diagnosis of prostate cancer or eating the right foods; and (2) knowledge of increased risk of prostate cancer for Black men. Participants believed it to be vital for Black men to be aware of their increased risk and, how to take action, for example, by making a GP appointment and feeling empowered to discuss their risk.

“I think we first need to be empowered with the information and the understanding to be able to present to a surgery, to a nurse to say, “This is what I believe. I want you to investigate this line.” Because we can present the evidence for ourselves. Then we can’t be blocked or fobbed off because we can say, “Well, you know, these are what the symptoms are. This is what I’m experiencing, so I believe it’s worth looking at that aspect.” (Male 6, aged 55–59, British Caribbean).

#### Other priorities- lack of time as a pragmatic barrier

Some participants mentioned not having time to access healthcare as a pragmatic barrier to prostate cancer health checks, as well as not having time to learn about healthcare subjects resulting in a lack of knowledge of prostate cancer.

‘Recognising the signs [of prostate cancer] bring me back to my first point when I was talking about information. We’re all working all the hours in the world, so we might not know the signs. So, that’s recognising the signs is probably bringing onto information, again.’’ (Male 9, aged 45–49, Ugandan).

#### Communication

Participants shared that having access to prostate health information in different languages is important. However, they highlighted this was not just about the English language but also about the cultural connotations connected to their community’s language. They noted it was vital that Black men feel the language used in prostate cancer information applies to them and that they can connect with the language or words used.

“Yes, because our language plays a lot, because language plays with a lot of stereotypes. For example, you know, language also promotes the skepticism, it promotes, also, the idea of traditions that people say, “This is not for us. This is not something that will happen for us.” Cancer, prostate, HIV, all of that, language plays into the mentality of having to raise something. So, you might be seeing it in English, and then say, “Oh, no, that’s for English,” you’d be like, “It’s a White man’s language, so that is it’’ (Male 7, aged 45–49, Cameroonian).

Making sure participants could also express themselves in a manner that a GP understands, was also highlighted as a barrier. Participants reported that some Black men lacked self-efficacy and assertiveness, which they believed necessary to be heard or taken seriously by a healthcare provider. They explained they had been brought up not to appear rude, and that assertiveness was perceived as rude in some African cultures, preventing them from speaking up as adults.

“Also, with the way the white man thinks is somehow a little bit different. The way we are brought up in Africa is that we are not brought up in a way where we are supposed to express ourselves. We are being subdued somehow. To me, I think that is a big problem.” (Male 4, aged 45–49, Nigerian).

“When you meet your own local people, you know how you can communicate to them. They can see reasons with you, they can understand you. I mean, you don’t expect an Indonesian person to understand someone who comes from Gambia. It’s going to be very difficult. So, I think, language has got to be one thing we’ve got to work on”. (Male 12, aged 45–49, Nigerian)

### Motivation

#### Perceived risk and fear of outcome

Some participants described their perceptions of prostate cancer risk to be a facilitator of early diagnosis of prostate cancer. Being aware and understanding statistics about increased risk for Black men, contributed to this. Participants also described associating the term ‘cancer’ more with women, and prostate cancer with age and specifically with ‘older people’.

“Because for me, most African men think cancer, it’s more of women, they don’t talk about [cancer] themselves.” (Male 4, aged 45–49, Nigerian).

Participants discussed their perceived risk of prostate cancer changing as they mature and grow older themselves.

“I need to be as informed as I can be about it, because I am getting to that age, which I thought was far away. But I’m there now.” (Male 3, aged 55–59, Zambian).

Not wanting to know and the fear of hearing the outcome of prostate cancer testing, were also mentioned as barriers to early diagnosis.

“And then you’ve got to drag yourself to the professionals, who’ve got to confirm to you what you really don’t want to confirm or otherwise.”(Male 9, aged 45–49, Ugandan).

#### Emotions due to negative experiences of healthcare-Experiences of institutional racism

Participants expressed they believed Black people are treated differently in the health system, resulting in barriers to accessing healthcare for prostate cancer health checks. They described an inequality in access for Black Caribbean and African communities. They shared feeling discriminated against when accessing healthcare and having to justify and fight for access to a GP or treatment. One participant described that the perceived racism was difficult to pinpoint but explained that at times the same medical standards did not seem to be applied to Black people and only to White people. For example, the participant opined that healthcare providers’ questions regarding medical conditions visible through changes to skin colour, are written for White people and do not apply to Black people, such as whether one is not feeling well because one looks pale. It was believed then that conclusions about their state of health were drawn based on standards that did not apply to them, but to White people.

‘I think for Caucasians, if they get ill, you might be able to tell from the colour of their skin. Some of the questions you go through if you’re doing an online assessment is, “Does the person look blue or purple?” or whatever colour. But none of those colours applies to me, so if you look at me, I’m the same colour whether I’m sick or healthy. So, if you come in and this person looks at you and says, “Well, on balance, you don’t really look sick.” (Male 5, aged 45–49 Ugandan).

Some participants stated they believed the inequality of treatment was more related to GPs’ ‘*ignorance*’ and lack of awareness regarding health issues for Black men, than to racism. Educating healthcare providers and GPs regarding health issues specific to Black people was suggested by all and was seen as essential to support men in accessing healthcare for prostate cancer health checks.

“I think they have been really complacent about [education of healthcare providers] despite some of the things that have happened to our communities and other minority communities within the health system for decades. I still think that they don’t do enough to actually understand the people that they are treating, whether that be language, whether that be cultural issues.” (Male 1, aged 45–49, Nigerian).

#### Emotions due to negative experiences of healthcare-Lack of trust

Some participants discussed that they did not understand why Black communities are targeted in terms of prostate cancer and other health promotion efforts. They indicated they did not trust statistics suggesting Black people were affected more by specific public health issues and were suspicious of these data.

“It doesn’t seem to be like there is a logic behind this targeting, all this targeting, like prostate cancer is affecting more Black people. I don’t understand. Maybe I need to educate myself and to enlighten myself here in this sense of getting the information from people in the medical field area, just to learn and to trust the medical person that is teaching us or giving us this guidance.’’ (Male 8, aged 50–54, Sudanese).

One participant stated that Black people do not trust ‘*the system’* in the Western world, linking this to governments and corruption in Africa. This was then also linked to trust in the GP. It was stated that the lack of confidence and trust in one’s GP impacted Black men’s decisions to access healthcare and acted as a barrier to accessing healthcare for early diagnosis of prostate cancer. Some participants related their feelings of suspicion and lack of trust to intrusive questions being asked as part of accessing care and their medical examination.

Lack of trust was also related to the personal connection participants felt with their GP. Trust, they felt, was built through personal connection. It was compared to building a relationship with their children’s school. Lack of trust was linked to the lack of time they were afforded with their GP and the feeling of not being taken seriously. Trust is not instant and getting to know each other was perceived to be essential to sharing personal and intimate information.

“I need to have a connection with my GP. Just like I have a connection with the school my children attend. They need to be able to put a face to my name. Now if these people don’t put a face to my name, I’m just a statistic. It becomes very difficult for both of us to be able to relate to each other.’ ’(Male 3, aged 55–59, Zambian).

A GP’s ethnicity, participants explained, was not important if building a personal connection was possible and there was trust, although some shared that this might be easier with ‘*someone who looks like you’*. The GP’s gender, however, did seem to matter to some and some participants explained that this was particularly linked to sexuality and embarrassment by potentially feeling aroused when examined by a female, particularly regarding intimate areas of the body such as the prostate. The GP’s age also mattered, and it was expressed that older generations might not want to be examined by younger doctors.

### Environmental influences-Opportunity

The theme of environmental influences refers to the opportunity participants have to engage with early diagnosis and consists of physical and social factors. Physical factors include access to healthcare. Social factors include cultural beliefs, social stigma, community endorsement, and social support.

#### Access to healthcare

A few participants reported that they had no issues accessing healthcare and they seemed to have positive experiences visiting their GP. However, the majority stated they experienced barriers to accessing healthcare and shared negative experiences, including challenges with navigating the healthcare system. Participants discussed that making appointments is challenging, highlighting the lack of screening programmes for prostate cancer. They compared this to established screening programmes such as colorectal screening, as well as breast and cervical cancer screening programmes for women and wished there would be organised screening programmes for prostate cancer, including invitations. Participants also talked about having access to annual health checks and discussed the inconsistency in service provision between practices and locations. Again, participants discussed active invitations that would facilitate access and wondered whether prostate exams could be included in annual health checks.

“Getting a doctor’s appointment, and then don’t forget large swathes of our particular community who are not even registered with a doctor.’’ (Male 1, aged 55–59, British Caribbean).

“It’s just that apathy to deal with the medics and the GPs, and all the stuff that you have to go through to get their attention.’’ (Male 5, aged 45–49, Ugandan).

“So, first all of, in Africa, we do tests every year. I go to my doctor and do a lot of tests on sugar, and this and that. But here it is so difficult. I have not been able to do it. […] But since I’ve been here, I’ve not been able to get an assessment with the GP.” (Male 11, aged 45–49, Nigerian).

#### Gatekeepers and not being taken seriously

Participants vehemently discussed experiencing GP receptionists as gatekeepers who they perceived as deliberately trying to create barriers for them to access GP appointments, for general and prostate health. Participants shared that receptionists made them feel like they had to justify being seen by a GP and that they are made to feel like they were ‘*time-wasters’* or a ‘*bother*’ by receptionists. Triage questions asked specifically by receptionists, as non-healthcare providers, were perceived as intrusive, and participants reported feeling uncomfortable, grilled, or belittled, which discouraged some participants from accessing healthcare. Participants discussed that some of these questions made them feel they were ‘*at fault’*. Some recounted being put off by these questions and experiencing these as barriers to contacting the GP practice the next time, or not attending the appointment for this very reason.

“All this screening on the phone made me feel a bit like maybe I don’t have to do that. I can tell you, categorically, I did not attend the first meeting I was supposed to have with the GP, just because I just felt like it was too much.’’ (Male 7, aged 45–49, Cameroonian).

“With the receptionists, I think, you know, it’s almost, though you have to prove to them that you are sick before they want to give you an appointment.’’ (Male 6, aged 45–49, British Caribbean).

Participants also described experiencing barriers to accessing healthcare in general due to not feeling listened to or being taken seriously by the GP. Participants felt this to be potentially underpinned by the short time slots GPs have to see patients, and they shared feeling rushed by these and described therefore not feeling heard. Participants viewed these short time slots within the context of the financial constraints the NHS operates within. They also discussed believing they were denied access to health checks due to *‘cost-cutting’* within the NHS and GPs having to make decisions about patients’ healthcare based on the practice’s budget.

“But he made mention of something that I was shocked. […] He was asking me why did I have to come and check my blood pressure? Because there is no issue with it. That was a long time ago. He made mention that, “Are you coming to do this because it’s free?” (Male 10, aged 45–49, Nigerian).

“Every time I’ve been to the GP, it’s like, I’m never taken seriously. I look healthy and they see no reason for me to be investigated for anything that I go there for.’’ (Male 3, aged 55–59, Zambian).

#### Cultural and religious factors

Participants also talked about cultural and religious beliefs in relation to prostate cancer health checks. They opined that talking about intimate and sensitive issues, such as prostate cancer, is difficult for Black men, as well as speaking up and that there was still a social stigma around this. Participants related sexuality and manhood to the challenge of discussing intimate issues as they believed that Black men have been raised to feel ‘*super*’, which makes showing weakness and vulnerability difficult. One participant also felt that certain words or subjects are more difficult to discuss because of their cultural meaning, such as death.

‘if you say it in a parable, in one of the African languages, it would be like, “Death is not for the people that are alive.” So, you can’t speak about death, because you are alive. I’m just giving an example.’’ (Male 7, aged 45–49, Cameroonian).

‘in African culture the fact is there are certain things you don’t talk about. […] issues to do with sexuality and death are things that you don’t just talk about anyhow.’’ (Male 3, aged 55–59, Zambian).

Participants did share that social change was occurring, which normalised having such conversations. This was related to being in a part of a world where health is talked about differently and health information is more accessible. However, one participant stated ‘*White folk think differently’*, indicating learned cultural behaviours are difficult to change and could lead to Black men being misunderstood. Generational change was, however, associated with younger Black men finding it easier to talk about intimate issues.

“I think another barrier for older men [is] social stigma, for the younger generation. Because the more the young generation [.], they have got connections. Some of the older men, black men, they were not born here, so they still have that [link] than the ones who were born here or maybe they’ve been here for a while so they’ve got an easier connection.” (Male 13, aged 50–54, Congolese).

Faith seemed to work both as a barrier and a facilitator to accessing prostate cancer health checks. Participants thought some Black people misinterpreted religious messages, which can make faith a barrier, such as praying rather than acting or accessing healthcare, ‘*if you pray it will go away’*. However, participants believed this is not what the Christian bible intended. Participants discussed that faith could work as a facilitator and encouraged them to look after their health. Some believed that their faith supported healthcare providers by giving them wisdom, explaining that God wanted them to live, not die and therefore they prayed for healthcare providers and the treatment they provide. Medical treatment provided was the process by which God heals, some participants shared. However, participants also discussed that there could be a conflict between medicine and the church and that some religious leaders encouraged their members to use prayer to heal. Other religious leaders supported seeking healthcare and involving religious leaders in health promotion was perceived as important to all.

“I’m a Christian and [.] you know, medical science, it works and God also works, and prayer does work. But, I have to know what I’m praying for. But some people buy into the doctrine of absolutely leave everything to God. [.] Some of the churches don’t even promote you going to the GP for a check-up. Because, you know, just pray. You know, it will happen. “You’ve got cancer? It’s going to disappear.” (Male 7, aged 45–49, Cameroonian).

“I am one person that believes that God does not want me to die for him. He wants me to live for him. Therefore[…], I pray for the doctors. I pray for the nurses. I pray for the medication that I’m going to take and that it should be able to work, because God wants me to live, not to die.’’ (Male 2, aged 50–54, Zimbabwean).

Participants discussed that traditional medicine was often used as a first-line of treatment, particularly for minor ailments. Although none of the participants used traditional medicine instead of western medicine, one participant stated he believed some groups would, in combination with prayer.

“For me, my religion doesn’t forbid me from seeking medical attention. But I know that there are some religious groups that do not believe in taking medication. They believe in taking some anointed oils. They believe in prayers. They may not seek medical attention because they believe, ‘If my pastor prays for me that disease will go away. It’s a matter of me having faith. If he prays then the disease goes away.’” (Male 2, aged 50–54, Zimbabwean).

Participants also discussed community endorsement and social support as facilitators. Some participants described relying on health advice from friends and relatives. They shared experiencing a level of trust and comfort from interacting with people from their own community, described as predominantly Black. They explained feeling more comfortable with having discussions about sensitive issues with their *‘own people’*.

They then also indicated that health promotion efforts would be more valuable if these came from members within the Black community, as these would be trusted. Participants recognised that their voices are valued and trusted in their community. They emphasised that community and religious leaders have an important role to play in creating awareness and tackling social stigma regarding sensitive health issues, such as prostate cancer. They felt that men supporting each other in overcoming embarrassment and social stigma would be helpful.

“It’s about how do we create awareness in our local churches. Because in our community, we trust our pastors more. So, now the question is, ‘How do we now get our pastor to understand what we are saying?’” (Male 10, aged 45–49, Nigerian).

#### The role of women

The role of women in prostate health communication was also perceived as a facilitator. Some participants highlighted the importance of discussing the topic with partners but it was also suggested to include women specifically in prostate health promotion. It was argued that women are better at ‘*organising*’ and therefore they could prompt men to act. In addition, it was stated that women would not hesitate to seek medical care and would not see help-seeking as a sign of ‘*weakness or a failing*’.

“But I think, if anything is bothering you, if you can’t share it with your wife, then I think it would also be very difficult for you to even share it with your doctor. So, I think it’s depending on the kind of relationship you have with your wife.” (Male 12, aged 45–49, Nigerian).

“I think, in my case, I am prompted, more often, by my wife, “Don’t miss your GP appointment. Don’t miss that.” some of the things that I just take for granted, my wife, she is particular about that. She will remind me constantly, and I think women should be involved in such issues, and we will see a result, I think.” (Male 2, aged 50–54, Zimbabwean).

However, others perceived that it was not the woman’s place to interfere with a man’s health and argued that this view would be more common among older generations, and aligned with traditional African culture. All agreed, however, that staying healthy for one’s family, particularly one’s children, was an important facilitator.

“For me, we need to contextualise because as I said, the generations are different, I do believe that, within the African community, women, they cannot [go straight] to the husband, we have to realise. It’s not that. I mean, I know that some of the people will say, “That’s African men,” but the way of African culture.” (Male 13, aged 50–54, Congolese).

In summary, the findings showed that participants perceived individual and environmental factors to influence help-seeking behaviour and the early diagnosis of prostate cancer among Black men. Key barriers included lack of knowledge and awareness, cultural and communication challenges, negative healthcare experiences, and systemic issues such as access to healthcare. Facilitators included the importance of community and faith-based support and the need for culturally sensitive health education to empower Black men towards early diagnosis and engagement with healthcare services.

## Discussion

Black men face a higher risk of prostate cancer; therefore investigating barriers to seeking early diagnosis is important to address this health inequity. This qualitative study explored barriers and facilitators to accessing healthcare for early diagnosis of prostate cancer among Black men in North-East England and Scotland. Although there is some US literature on this topic, there is a dearth of literature from the UK. Our findings indicated that barriers and facilitators were complex and multifaceted, in line with the I-SAM [28]. Barriers at individual level include a lack of awareness and knowledge of the risk of prostate cancer, a common barrier widely reported [15, 16, 29]. A lack of in-depth knowledge of prostate cancer included knowledge concerning susceptibility and thinking prostate cancer was for older men only, not knowing symptoms, not being aware of the increased risk for Black men but also not understanding the lack of a prostate screening programme. The absence of such a programme was received with some dismay as participants compared this with existing UK screening programmes (e.g. breast, colorectal and cervical). Black men have been found to lack knowledge regarding symptoms of prostate cancer as a consequence of the challenges they experience to seeking health information [20].

Health promotion efforts to increase knowledge and raise awareness of the risk of prostate cancer through health education are crucial; however, the complexity of the barriers suggests that health education, raising awareness, and possessing knowledge alone are not enough. Communication barriers and language issues were identified. Health literacy is an important barrier to engaging with and accessing healthcare [30]. Therefore, ensuring that prostate cancer communication is clear and accessible to all is key. Importantly, participants indicated that if they could not identify or connect with the language or words used, they did not pay attention to the message. Meaning-making relies on language. Perceptions of a lack of shared language can result in reduced interaction with healthcare [31]. Ensuring that prostate cancer communication is developed in partnership with the target communities, would help overcome this barrier and ensure that the language used is relatable.

Participants expressed that sources of health communication were often informal and they relied on friends. A systematic review regarding sources of prostate cancer communication for Black men, corroborates this finding and indicates that print materials are not ideal for communicating prostate cancer information and that Black men preferred to find out about prostate cancer from familiar individuals such as family, partners, or community and religious leaders [32]. The emphasis on wishing to hear about health issues, such as prostate cancer, from familiar and trusted sources links to the lack of trust in the healthcare system that participants reported, which has also been reported in the literature [15, 32, 33]. Although some participants shared positive experiences with healthcare, many shared negative stories. These previous negative experiences deterred them from accessing healthcare for prostate health checks. Not being able to build a personal connection with their GP was a significant factor, and the lack of a trusting relationship with health professionals has been found to be a barrier to help-seeking [15]. Navigating the healthcare system and finding it too difficult to make an appointment was discussed, which has been reported previously for Black men in the UK [20, 34]. Dealing with triage questions they perceived as intrusive, resulted in some men describing not attending a GP appointment after such a phone call.

Asset-based approaches that use a community’s assets such as networks or community cohesion, can be effective tools in health promotion to empower communities and build social capital [35]. Such approaches include participatory methods and working in partnership with the community, using community champions or peer-to-peer support, and offering useful tools [36]. For example, trusted members from the community or Black healthcare providers could explain why triage questions are asked and practise with the community how best to deal with these. Participants discussed not feeling heard by the GP, partly due to experiencing pressure from being afforded limited time with their GP and concern about wasting the doctor’s time, findings that are supported by the literature [37]. Participants did not know how to deal with this, and some stated they lacked assertiveness out of concern for appearing rude. Offering prostate cancer awareness training in informal settings guided by peer-to-peer support or the use of lay communities or peer educators in prostate cancer communication can offer trusted support [38]. Training could include navigating the healthcare system, practising communication with healthcare providers or offering tools to the Black community, for example in the form of questions to ask the GP. These tools could all help with communicating with the GP [39]. Communication between doctors and patients is a core component of the patient experience and Black and Asian ethnic minority groups have reported lower satisfaction with the quality of communication than the general population [40, 41], as well as with the NHS [42].

Negative experiences also included perceived discrimination and racism. Racial bias has been reported as a barrier to accessing healthcare [6, 43, 44]. A 2022 survey with 2051 Black or mixed back ethnicity people in the UK found that 65% of respondents had experienced discrimination from healthcare professionals [45]. A report by the NHS Race Health Observatory supports findings of institutional racism and cultural insensitivity across healthcare services for Black and ethnic minority groups [46]. Including cultural humility training in medical education is essential to tackling these issues. Cultural humility embraces a mindset of continuous learning, prioritising curiosity and self-reflection when interacting with individuals from diverse cultural backgrounds. It highlights the importance of acknowledging patients’ cultural perspectives as equally valuable and encourages critical contemplation of how structural factors and power dynamics influence healthcare. By respecting patients’ cultural values and life experiences, this approach has the potential to transform power dynamics and create a more inviting and inclusive healthcare environment, fostering patients’ willingness to participate actively in their healthcare [47]. Cultural humility training for healthcare and support staff developed in partnership with ethnic minority groups, might be valuable to ensuring this issue is tackled urgently and is practised across the healthcare system.

Cultural factors also emerged from the data. Participants mentioned social stigma and a reluctance to discuss health issues with others, which has also been reported in the literature [20]. The difficulty for men in discussing these sensitive issues, such as prostate cancer, related to not wanting to appear vulnerable or weak, which was linked to preservation of manhood which has been reported in other studies [29, 48]. Tackling the social stigma and normalising conversations about health and sensitive issues like prostate cancer, needs to come from within the community. Involving trusted members of the community, such as family, community and religious leaders, and Black healthcare professionals, in prostate cancer communication will therefore be essential. The collectivist nature of Black African and Caribbean communities, where individuals see themselves primarily as part of a larger social network, can be a powerful asset in health promotion. For example, participants spoke extensively about the role of women in encouraging men to engage with their health, and staying healthy for the sake of children and family was seen as a strong enabler to help-seeking. Testimonials from Black prostate cancer survivors could also be used to stimulate awareness and improve early help-seeking behaviour.

Ensuring health promotion is culturally tailored and relevant is important. Religious factors could be part of cultural tailoring. In this study, religious factors emerged as both barriers and as facilitators. Fatalism appeared to be a barrier for some, when praying, rather than accessing medical care, was practised or encouraged. However, more participants indicated that their interpretation of their faith stimulated engagement with medical care. In the US, church-based settings have been used for prostate cancer education, but few studies have used faith-based approaches. Using messages that are congruent with individuals’ spiritual beliefs is another example of an asset-based approach and a useful form of health promotion in cancer communication [32, 49], which has been successfully used in prostate cancer communication specifically [50].

### Limitations

A limitation of our study was that the sample was small, self-selected, relatively young and highly educated, although most participants had only limited knowledge of prostate cancer. While we aimed to recruit a diverse sample, all men identified as Christian and Black men with different religions should be included in future work. We did not collect data on participants’ postcodes or other social determinants, which limits our ability to explore geographic or socioeconomic variation in views and experiences. This is an important consideration given known inequalities in prostate cancer diagnosis across the UK, including the North–South regional divide, and should be addressed in future research.

Conducting focus groups online has potential limitations in terms of the flow of the conversation [51]; however, this method allows for the inclusion of men from two UK regions, thereby enhancing the applicability of our findings. Transferability of findings to other countries may be limited due to differences in healthcare systems.

Strengths included the serial focus groups and iterative analysis, which allowed issues to be explored in more depth when needed. The member-checking exercise where findings were reported back to participants also added to the study’s rigour. Although the initial two focus groups were led by a White male researcher, supported by two Black researchers, in the final two focus groups the team was entirely Black. Ethnic matching of interviewer and interviewees could be argued to increase a source of bias [52], although here it was believed to build trust [53] and the data were analysed by researchers not involved in data collection, decreasing the chance of bias.

## Conclusion

This qualitative study with Black men in North-East England and Scotland found a complex web of barriers and facilitators to accessing healthcare for early diagnosis of prostate cancer. The complexity of these barriers indicates that health education alone is not enough. Asset-based and participatory approaches that allow working in partnership with the community are needed to develop interventions to tackle these barriers and use facilitators to increase awareness of the risk of prostate cancer and encourage early help-seeking behaviours. Future work will require a dual focus: tackling barriers to early diagnosis of prostate cancer within the healthcare system, and working in partnership with communities. Structural barriers to accessing healthcare and experiences of racism need to be addressed within the healthcare system, including through cultural humility training for healthcare and support staff, informed by ethnic minority groups, and proactive support from GPs to help higher-risk men engage in discussions about prostate cancer health checks and improve communication. Further research in partnership with Black African and Caribbean communities is needed to develop asset-based and culturally relevant interventions to build trust and encourage engagement with the healthcare system, to increase awareness of the risk of prostate cancer and encourage early help-seeking behaviour for Black men.

## Supplementary Information

Supplementary Material 1.

## Data Availability

Anonymised data can be made available upon reasonable request by contacting the corresponding author.
