# Supplementary material for: Barriers and facilitators to accessing healthcare for early diagnosis of prostate cancer for black men—a qualitative exploration in North-East England and Scotland
Source: BMC Public Health. 2025 Jul 14;25:2454. doi: 10.1186/s12889-025-23650-y (PMC12257739; doi:10.1186/s12889-025-23650-y)
Supplement: Supplementary file 1 — Supplementary Material 1. [file 12889_2025_23650_MOESM1_ESM.pdf]

TOPIC GUIDE FOR EXPLORATION OF BARRIERS AND FACILITATORS TO EARLY DIAGNOSIS OF PROSTATE CANCER

Introductions

- Welcome and thank you
- Introductions – Research Team and Research Participants
- Please do let us know, tell us, type in the chat box or wave at us if you can't hear if anything or something is not working. This person x [member of the research team] can help you with any IT issues.
- Can you use the reaction buttons to give a thumbs up if everything is fine?

Aim of the research and PICE group

We are delighted that you are here today and we are hoping you will become part of our PICE group, this stands for Public Involvement and Community Engagement group. This is a group that advises us throughout the whole 2 year the research takes place.

Black men are twice as likely to develop prostate cancer as white men. Early diagnosis can save lives, but black men are often diagnosed late. The aim of the research is to develop a workshop for black men to raise awareness of the risk of prostate cancer for black men. We need to make sure that the workshop is developed by black men, for black men. So your voice and input in the research is incredibly important.

Before we can develop the workshop, we need to understand together with you what might make it difficult for black men to seek help regarding prostate cancer. Therefore, the aim of these two focus groups is to explore with you what are the things that might make it difficult for you or for other black men to see the doctor regarding prostate issues, and what might make it easier to go and see the doctor. We will do this over two focus groups, today and the next meeting we will plan for in the next few weeks. We will start exploring these issues today and can dive into some specific issues in more depth the second time we meet.

Once we have a clearer idea after these first two focus groups what are the things that might make it difficult for black men to see the doctor regarding prostate issues, we want to develop with you the workshop to raise awareness. This would take approximately three more online meetings. We would then also want to train some of you to help us to deliver the workshop and we would do this in a further three meetings. You will receive a gift voucher for each meeting.

Does anyone have any questions about this or anything relating to the research?

- Confirmation from each participant to have received the participant information sheet and also e-signed the consent form.

- Short icebreaker to make everyone feel comfortable and get used to all contributing.

- Confirm with everyone they have consented to recording the session

Ground Rules

Let's get started with the first focus group then. To make sure this can run smoothly we think it is a good idea to set some ground rules.

Everyone will have the chance to participate and express their views during this focus group session. Everyone may have a different opinion; we are all entitled to our own ideas and thoughts, and we want to hear ALL of them. Please can we abide by the following ground rules for the smooth running of this focus group.

- To be polite and respect each other's opinion and thoughts at all times
- Not to be disrespectful towards anyone or laugh at other people
- There are no right or wrong answers, so don't worry about saying a wrong thing; we might also inquire further just to be sure, so we just don't assume things.
- To speak openly and honestly about your views and opinions (your comments are completely confidential, and your name will not be used in any report).
- To keep other people's comments confidential, by not sharing them with people outside of this group. This means whatever we say here stays here.
- To try to speak clearly and one at a time, for good audio recording and subsequent transfer to paper.
- The moderator may have to interrupt our comments at times, we're sorry to interrupt you, but we want to make sure we can cover all the topics and also, we can finish at the stipulated time. We don't want to keep you at your screens for too long.
- If you do need to leave early unexpectedly, that is okay. Taking part in our meetings is voluntary, so you can leave at any time you want, and you don't have to answer any questions or talk about anything you don't want.
- Some of your views and opinions might be about how **you** feel personally, or how you think **black men** feel or how you think **most men** feel; please do let us know which one you are talking about when you share your ideas.
- Please let us know if you're ready to start.

TOPIC GUIDE

1. To start with, what motivated you to join this research?

Knowledge/attitudes

1. What do you think or feel when you hear 'prostate cancer'? How do you think other black men think/feel about prostate cancer?
2. What do you know about prostate cancer? (probe: is prostate cancer a serious disease? Do many men get prostate cancer? What about black men? Can you live with prostate cancer? Does one die of prostate cancer?)
3. Do you think some groups of men may be more likely to get prostate cancer? (probe: different age groups? Family? Do you think you are at an increased risk? Black men?)

4. What do you think might be the symptoms of prostate cancer?
5. Have you ever heard about tests to check for prostate cancer?

Sources of Information

6. Where do you go for information about your health?(probe: if participants do not seek health information, probe to explore that; are there any differences in health seeking?)
7. Where might you go for information about cancer specifically? (probe for similarities/differences when seeking general health information vs cancer information. If participants do not seek health information, explore that too).
8. Where might you go for information about prostate cancer? (probe: have you ever searched for information? How was that? What was helpful? What was not?)
9. Has any doctor or nurse ever spoken to you about prostate cancer? (probe: Who? What was that like?)

Help seeking attitudes

10. How do black men feel about accessing healthcare?
11. How do you feel about accessing healthcare? (probe: do you go to the doctor? What might prompt you to go to the doctor? When might you go to the doctor? Do you engage in preventative healthcare, e.g. annual health check? What has been your experience in accessing healthcare?)
12. When do you think men should go and look for help regarding prostate cancer? (probe: should this be at a certain age? Preventative? Should this be when experiencing symptoms?)
13. What would you do if you experienced symptoms of prostate cancer? (probe: for example what would you do if you experienced having to pee more frequently, or pee during the night? Difficulty peeing, difficulty in starting to pee? Probe: where would you go if you would experience any symptoms? doctor/GP/nurse? Anywhere else? Friends/partner/family?)
14. How do you think men might feel going to the doctor to talk about prostate cancer symptoms?
15. How do you think black men might feel going to the doctor to talk about prostate cancer symptoms (probe: similarities/differences with others? white men? women?)
16. What do you think might make it difficult for someone to go to the doctor to talk about prostate cancer symptoms? (probe: what do you mean by that? Can you explain that more? What do others think? Any other reasons why it might be difficult?)
17. Is there anything that makes it difficult for you to go to the doctor to talk about prostate cancer symptoms?
18. Is there anything in particular about the sensitive and intimate nature of prostate cancer do you think that is difficult to talk about for you? (probe: what about for black men?)

19. In the literature it is sometimes described that men have a fear of the procedure of the prostate checks. How is that for you? (probe: what about these tests might be difficult? and what do you think might this be like for other black men?)
20. In the literature it is sometimes described that men link these checks with their sexuality and that they might feel threatened in terms of their manhood. How would that be for you? (probe: and for other black men?)
21. In the literature it is also sometimes described that men have a fear of the outcome of the tests. How is that for you? (probe: what do you think might this be like for other black men? What would it be like to have a diagnosis of prostate cancer? How might this be perceived in the black community? What would it be like to have treatment for prostate cancer?)
22. Is there anything that would make it easier for you to look for help with prostate cancer? (probe: and for other black men?)

Healthcare providers

23. How do you feel about your GP or nurse? (Probe: Do you feel comfortable with them? Do you trust them?)
24. Do black people feel comfortable with their GP or nurse? (probe: Why or why not? What would need to change?)
25. What could GPs or nurses do to make it easier for you to discuss your health?
26. What could GPs or nurses do to make it easier for you to discuss prostate cancer?
27. If your doctor recommended you to have prostate cancer checks, would that be important to you?
28. Does the gender of the GPs or nurses matter to you?
29. Does the ethnicity of the GPs or nurses matter to you (probe: would you prefer it if they were black? Why or why not?)

Social and cultural factors

30. Do you discuss health issues with others? (probe: why or why not?).
    a. How do you feel about discussing health issues with other men?
    b. How do you feel about discussing prostate cancer with other men? (probe: how is that for you?)
31. Do you discuss health issues with your family or your partner? (probe: why or why not?). How do you feel about discussing health issues with your family or partner?
    c. Do you discuss cancer with your family or partner?
    d. Do you discuss prostate cancer with your family or partner? (probe: How is that for you?)
32. How do you think discussing issues such as prostate cancer is perceived in the black community? (probe: is this difficult? If so, why? How could change be achieved if change is needed?).
33. Do you think there are particular beliefs regarding cancer and prostate cancer in the black community that are helpful or unhelpful for black men? (probe: what do you mean by that? What do others think?)

34. What do you think could be the role ofwomen in raising awareness of the risk of prostate cancer? (probe: do (black) women find it difficult to talk about these issues, could they support men, how?)
35. Do you attend black community organisations, for example church? Are health issues discussed there? (probe: what's that like? Is there a role for community organisations to promote health issues? What about prostate cancer? Is there a role for church leaders?).
36. Do you know anyone with prostate cancer? (probe: does that change how much you understand of the disease? How you perceive it?)
37. Do you think there is a role to play for other members of the black community in raising awareness of the risk of prostate cancer in the black community? (probe: barbers/hairdressers?)

Is there anything you would like to add that we haven't discussed yet?

Thank you so much for taking part in the focus group. Share details next meeting.

TOPIC GUIDE FOR EXPLORATION OF BARRIERS AND FACILITATORS TO EARLY DIAGNOSIS OF PROSTATE CANCER (Focus group 2)

Introductions

- Welcome and thank you
- Introductions – Research Team and Research Participants
- Please do let us know, tell us, type in the chat box or wave at us if you can't hear if anything or something is not working. This person x [member of the research team] can help you with any IT issues.
- Can you use the reaction buttons to give a thumbs up if everything is fine?

Aim of the research and PICE group

Thank you all so much for coming back.

Quick reminder of the purpose of the research and the PICE group-so we're hoping that you and us will develop an intervention/workshop together, in partnership, to tackle some of the challenges black men face that may prevent early diagnosis of prostate cancer. But before we can do that, we need to understand better what those challenges black men face are, and that is the purpose of these first two focus groups. So it's great that you here, thank you. We made a really good start last time and we're hoping to dive in some of these issues we discussed further today.

Does anyone have any questions about this or anything relating to the research?

We discussed the ground rules last time, just a quick reminder that everything we discuss here is confidential, so please do not share outside of the group. There are no wrong or right answers, and please do respect each other, as you all did last time.

- Confirmation from each participant to have received the participant information sheet and also e-signed the consent form.

- Confirm with everyone they have consented to recording the session

Please let us know if you're ready to start.

TOPIC GUIDE

Last time we discussed that some of you found going to the GP difficult. I wonder if we can explore this further.

Help seeking attitudes

38. How do black men/you feel about accessing healthcare? (broader context) (probe: do you go to the doctor? What might prompt you to go to the doctor? When might you go to the doctor? Do you engage in preventative healthcare, e.g. annual health check? What has been your experience in accessing healthcare?)
39. How do you think black men might feel going to the doctor to talk about prostate cancer symptoms?
40. What do you think might make it difficult for someone to go to the doctor to talk about prostate cancer symptoms?
41. Is there anything in particular about the sensitive and intimate nature of prostate cancer do you think that is difficult to talk about for you? (probe: what about for black men?)

Healthcare providers & Trust

We talked last time about trust, trust in the system, trust in healthcare providers. Let's explore that some more.

1. Do black people feel comfortable with their GP or nurse? (probe: Why or why not? What would need to change?)
2. What could GPs or nurses do to make it easier for you to discuss your health?
3. What could GPs or nurses do to make it easier for you to discuss prostate cancer?
4. If your doctor recommended you to have prostate cancer checks, would that be important to you?
5. Does the gender of the GPs or nurses matter to you?
6. Does the ethnicity of the GPs or nurses matter to you (probe: would you prefer it if they were black? Why or why not?)
7. Some of you mentioned inequalities and racism last time in healthcare. Let's discuss this some more. Have any of you experienced this?

Social and cultural factors

We discussed last time that black men find it difficult to talk about health issues and prostate cancer. Some you shared that you felt things were changing for younger generations. Let's explore that some more.

1. Do you think there are particular beliefs regarding cancer and prostate cancer in the black community that are helpful or unhelpful for black men? (probe: what do you mean by that? What do others think?)
2. What do you think could be the role of women in raising awareness of the risk of prostate cancer? (probe: do (black) women find it difficult to talk about these issues, could they support men, how?)
3. Do you attend black community organisations, for example church? Are health issues discussed there? (probe: what's that like? Is there a role for community organisations to promote health issues? What about prostate cancer? Is there a role for church leaders?).
4. Do you think there is a role to play for other members of the black community in raising awareness of the risk of prostate cancer in the black community? (probe: black men with experience of prostate cancer/ barbers/hairdressers?)

Fear

5. In the literature it is sometimes described that men have a fear of the procedure of the prostate checks. How is that for you? (probe: what about these tests might be difficult? and what do you think might this be like for other black men?)
6. This came up briefly last time, in the literature it is sometimes described that men link these checks with their sexuality and that they might feel threatened in terms of their manhood. How would that be for you? (probe: and for other black men?)
7. In the literature it is also sometimes described that men have a fear of the outcome of the tests. How is that for you? (probe: what do you think might this be like for other black men? What would it be like to have a diagnosis of prostate cancer? How might this be perceived in the black community? What would it be like to have treatment for prostate cancer? Would prostate cancer be a death sentence?)

8. Is there anything that would make it easier for you to look for help with prostate cancer? (probe: and for other black men?)

Is there anything you would like to add that we haven't discussed yet?

Thank you so much for taking part in the focus group today
